# No Impact of Probiotics to Reduce *Clostridium difficile* Infection in Hospitalized Patients: A Real-world Experience

**DOI:** 10.1093/ofid/ofy192

**Published:** 2018-12-13

**Authors:** Maggie J Box, Kristine N Ortwine, Miguel Goicoechea, Scripps Antimicrobial Stewardship Program (SASP)

**Affiliations:** 1 Department of Pharmacy, Scripps Health, San Diego, California; 2 Division of Infectious Diseases, Scripps Health, San Diego, California

**Keywords:** *Clostridium difficile* infection, hospitalized patients, probiotics

## Abstract

We assessed the effectiveness of a Lactobacillus probiotic on rates of health care facility–onset *Clostridium difficile* infection (HO-CDI) in patients receiving antibiotics. A total of 1576 patients were evaluated. There was no difference in the HO-CDI incidence between those who received probiotics and those who did not (1.8% vs 0.9%; *P* = .16).

Hospitals participating in the Centers for Medicare and Medicaid Services report health care facility–onset *Clostridium difficile* infection (HO-CDI) data to the National Healthcare Safety Network. The Society for Healthcare Epidemiology of America and the Infectious Diseases Society of America endorse recommendations to assist acute care hospitals in implementing and prioritizing their CDI prevention efforts [1]. Although probiotics is not an endorsed strategy, a recent supplement published in *Clinical Infectious Diseases* recommends probiotics, specifically the combination of *Lactobacillus acidophilus* CL1285, *Lactobacillus casei* LBC80R, and *Lactobacillus rhamnosus* CLR2 (Bio-K+), as an intervention to reduce CDI rates [2]. Based on data presented, our institution added Bio-K+ to the formulary, and as part of a “bundle” to reduce risk of HO-CDI, Bio-K+ was recommended to be administered to patients on antibiotic therapy identified as high risk for CDI. In addition, education was provided to medical staff regarding the availability of Bio-K+, and physicians could choose to administer Bio-K+ at their discretion. This study evaluated the rates of HO-CDI for a 6-month time period among patients who received intravenous (IV) antibiotics plus Bio-K+ vs IV antibiotics alone.

## METHODS

This was a retrospective cohort study conducted at a 400-bed community hospital in La Jolla, California. All hospitalized patients treated with IV antibiotics during the study period were evaluated for enrollment. Adult patients (age ≥18 years) who received at least 1 dose of antibiotics and had a length of stay >3 days were included. Patients were excluded if CDI was community onset (diagnosed within 3 days of hospital admission) or if they received cefazolin or cefoxitin for surgical prophylaxis only. The primary outcome was the incidence of HO-CDI in patients who received IV antibiotics plus probiotics vs IV antibiotics alone. Bio-K+ was the only probiotic used was and was prescribed at the discretion of the attending physician.

Baseline demographic data, length of stay, age, Charlson Comorbidity Index, billed grams of antibiotics, acid inhibitor use, number of days on probiotics, intensive care unit (ICU) stay, and in-house mortality were evaluated. Patients were identified to have received IV antibiotics if they received at least 1 dose of the following: vancomycin, ciprofloxacin, levofloxacin, ceftriaxone, ceftazidime, cefepime, piperacillin/tazobactam, imipenem/cilastatin, meropenem, ertapenem, cefazolin, or cefoxitin. Patients were considered to be on probiotics before the onset of HO-CDI if any doses of Bio-K+ were recorded before the date of *Clostridium difficile* toxin testing. The study was approved by the Scripps Institutional Review Board.

Descriptive statistics were used to analyze demographic data across the 2 cohorts. Continuous outcomes were analyzed by 2-tailed Student *t* test, and dichotomous data were analyzed by the Pearson χ^2^ test or Fisher exact test (for cell size <5). All statistical analyses were performed using R: A Language and Environment for Statistical Computing, version 3.0.1 (Vienna, Austria) [3].

## RESULTS

Between March 29, 2016, and September 30, 2016, a total of 1576 patients treated with IV antibiotics were evaluated, of whom 649 received antibiotics plus probiotics and 927 were treated with antibiotics alone. Both groups were similar with respect to age (65.8 vs 67.2 years; *P* = .15), ICU stay (48.4% vs 49.2%; *P* = .75), and in-house mortality (8.2% vs 6.8%; *P* = .32). HO-CDI occurred in 11 of 649 patients who received antibiotics plus probiotics and in 8 of 927 patients treated with antibiotics alone (1.8% vs 0.9%, respectively; *P* = .16) (Table 1). The median duration of probiotic treatment was 8.1 days. Patients in the probiotic group had a longer length of stay, a higher Charlson Comorbidity Index, and a higher amount of antibiotics billed (Table 1).

**Table 1.** Incidence of HO-CDI Between Patients Who Received Antibiotics

Outcome	Probiotic + ABX (n = 649)	ABX Only (n = 927)	*P* Value	Top 30% ABX Only (n = 284)	*P* Value
HO-CDI, No. (%)	11 (1.8)	8 (0.9)	.16	5 (1.8)	1
Age, mean (SD)	65.8 (18.7)	67.2 (18.6)	.15	64.8 (17.3)	.23
Length of stay, median (IQR), d	9 (6–16)	6 (5–9)	<.0001	8 (5–14)	.06
Charlson Comorbidity Index, mean (SD)	4.6 (3.4)	4.2 (3.2)	.011	4.3 (3.1)	.049
Billed g of antibiotics, median (IQR)	24 (9.2–52.2)	10 (5–19.8)	<.0001	34.5 (22.6–52.6)	<.0001
Acid inhibitor use, No. (%)	482 (74.3)	663 (71.5)	.25	210 (73.9)	.93
Average time on probiotics, d	8.1	—	—	—	—
ICU stay, No. (%)	314 (48.4)	456 (49.2)	.75	154 (54.2)	.10
In-house mortality, No. (%)	53 (8.2)	63 (6.8)	.32	32 (11.3)	.13

Abbreviations: ABX, antibiotics; HO-CDI, health care facility–onset *Clostridium difficile* infection; ICU, intensive care unit; IQR, interquartile range.

To evaluate whether greater antibiotic exposure in the probiotic group offset a potential therapeutic benefit, we conducted a subgroup analysis. We compared HO-CDI rates in the probiotic group with rates in the top 30% of patients by antibiotic exposure (billed grams of antibiotics) in the antibiotic-alone group and observed no difference (5 of 284 patients, 1.8%; *P* = NS) in HO-CDI rates (Table 1). Further, the high–antibiotic exposure group had a significantly greater amount of billed grams of antibiotics than the probiotic group (median, 34.5 vs 24.0 g; *P* < .001), despite similar lengths of stay (median, 8 vs 9 days; *P* = .06).

## DISCUSSION

In this study, we observed no difference in the rates of HO-CDI among hospitalized patients who received IV antibiotics, with or without probiotics. One notable difference, however, was that the probiotic group had greater antibiotic exposure, as measured by total grams of antibiotics billed during their hospital stay. We attempted to adjust for this difference by comparing CDI episodes with the subset of patients who were exposed to comparable or higher amounts of antibiotics as the probiotic group and, again, observed no difference in HO-CDI rates.

This study has limitations to consider. First, probiotic use was not standardized and was prescribed at the discretion of the attending physician. A systematic review by Shen et al. [4] found that probiotics were most effective if given closer to the first antibiotic dose, with a decrement in efficacy for every day of delay in starting probiotics. From our data, we are unable to determine the timing of probiotics relative to the first dose of IV antibiotics. Probiotics were administered using a standard dose; however, compliance was not assessed. Second, meta-analyses have shown a modest benefit in preventing first-episode CDI [4, 5], with no benefit on treatment or prevention of recurrence [6]. In this study, we evaluated HO-CDI and did not differentiate between initial and recurrent episodes. Third, probiotic use was not randomized and patients who received probiotics also had higher antibiotic exposures compared with those who did not. Physician bias regarding the efficacy of probiotics should be recognized and merits consideration if physicians with more confidence in probiotic efficacy have different antibiotic prescribing patterns. Additionally, we did not compare the distribution of classes of antibiotics administered between groups. Probiotic administration was not stratified by receipt of an antibiotic that would be associated with a “higher risk” of HO-CDI, and the goal of this study was to provide an overall “global” assessment of probiotic efficacy in our hospitalized population.

Despite these limitations, this study accurately describes probiotic use at our institution and may be more generalizable to real-world use of probiotics at other acute care hospitals. Meta-analyses demonstrating benefit of probiotics are heavily weighted on 2 small positive studies by Gao et al. [7] and Rafiq et al. [8]. These studies demonstrated very large effect sizes of Lactobacillus-containing probiotics compared with placebo (95% [7] and 75% [8] reduction in CDI, respectively) in populations with very high first-episode CDI prevalence rates (23.8% [7] and 40% [8], respectively). Our results and CDI prevalence are more consistent with the PLACIDE study and population [9]. This large, multicenter, double-blinded, placebo-controlled trial randomized hospitalized patients to a probiotic containing 2 strains of *Lactobacillus acidophilus* and bifidobacteria vs placebo and observed no difference in CDI rates (12 of 1470 patients [0.8%] vs 17 of 1471 patients [1.2%]; *P* = .35). Recent CDI guidelines affirm the need to use caution in applying study results with an abnormally high baseline incidence of CDI and conclude that there are insufficient data at this time to recommend probiotics for primary prevention of CDI outside of clinical trials [1].

Antibiotic use is the most important modifiable risk factor for CDI in acute care hospitals. The Centers for Disease Control estimates that at least 30% of antibiotic use is unnecessary [10]. Based on these findings, our institution removed all probiotics from the formulary. Instead, we endorse strong antimicrobial stewardship practices that are shown to be efficacious and caution that probiotics may consume health care resources without adding additional benefit.
